# Policy Implications for the COVID-19 Pandemic in Light of Most Patients (≥72%) Spending at Most One Night at the Hospital After Elective, Major Therapeutic Procedures

**DOI:** 10.7759/cureus.9746

**Published:** 2020-08-14

**Authors:** Richard H Epstein, Franklin Dexter, Todd J Smaka, Keith A Candiotti

**Affiliations:** 1 Anesthesiology, University of Miami Miller School of Medicine, Miami, USA; 2 Anesthesiology, University of Iowa, Iowa City, USA; 3 Anesthesiology, Univeristy of Miami Miller School of Medicine, Miami, USA

**Keywords:** covid-19, length of stay, patient discharge, elective surgical procedures

## Abstract

A large number of inpatients with Coronavirus disease 2019 (COVID-19) in some regions of the United States may interfere with the ability of hospitals to take care of patients requiring treatment for other conditions. Nonetheless, many patients need surgery to improve their quality of life and to prevent deterioration in health. Curtailment of services also negatively affects the financial health of hospitals and health systems. Broad policies to prohibit all “elective” surgical procedures to ensure that there is sufficient hospital capacity for pandemic patients may be unnecessarily restrictive because, for many such procedures, patients are rarely admitted following surgery or only stay overnight. We studied all elective inpatient and ambulatory cases involving major therapeutic procedures performed in the state of Florida in 2018. We mapped the primary procedure to the corresponding Clinical Classification Software (CCS) category. We determined the distributions of lengths of stay overall and as stratified by CCS category, then calculated the percentage of cases that had a hospital length of stay of ≤1 night (i.e., 0 or 1 day). A threshold of one night was selected because patients discharged home on the day of surgery have no effect on the inpatient census, and those staying overnight would either have a transient effect or no effect if observed overnight in the postoperative care unit. Among the 1,852,391 elective cases with one or more major therapeutic procedures, 65.2% (95% lower confidence limit [LCL] = 65.1%) of cases had a length of stay of 0 days and 72.9% (95% LCL = 72.8%) had stay ≤1 day. There were 38 different CCS categories for which at least 95% of patients had a length of stay of ≤1 day. There were 28 CCS codes that identified 80% of the patients who were discharged with a length of stay ≤1 day, showing representation of multiple surgical specialties. Our results show that even in the face of constraints imposed by a high hospital census, many categories of major therapeutic elective procedures could be performed without necessarily compromising hospital capacity. Most patients will be discharged on the day of surgery. If overnight admission is required, there would be an option to care for them in the postanesthesia care unit, thus not affecting the census. Thus, policies can reasonably be based on allowing cases with a substantial probability of at most an overnight stay rather than a blanket ban on “elective” surgery or creating a carve-out for specified surgical subspecialties. Such policies would apply to at least 72% of elective, major therapeutic surgical procedures.

## Introduction

The census of inpatients infected with the SARS-CoV-2 virus is high at many hospitals in the United States, interfering with the ability to take care of patients requiring treatment for conditions other than Coronavirus disease 2019 (COVID-19) [1]. Nonetheless, many patients need surgery to improve their quality of life and to prevent deterioration in health [2]. Furthermore, the curtailment of services has a substantive negative financial effect on hospitals and health systems [3].

Broad policies enacted by state or local governments to prohibit all “elective” surgical procedures to ensure sufficient hospital capacity for pandemic patients may be unnecessarily restrictive because they do not consider the lack of impact from many elective cases on the hospital census [4]. For example, there would be no rationale to restrict patients from having elective cataract surgery based solely on concern on the availability of inpatient beds because those patients are rarely admitted to hospitals following their procedure [5,6]. On the other hand, there could be a reason to restrict care based on other factors such as the absence of sufficient quantities of personal protective equipment, lack of availability of timely preoperative reverse transcriptase-polymerase chain reaction testing for SARS-CoV-2, or inadequate numbers of healthy medical personnel [7]. For example, in Florida, the executive order by Governor DeSantis that stopped all elective surgery was issued on March 20, 2020, because “… appropriate steps must be taken to conserve all medical supplies, including personal protective equipment...” [8].

We are unaware of any studies of the distributions of the length of stay for the broad range of surgical procedures performed at hospitals and ambulatory surgery centers [9]. However, procedures classified by the Current Procedure Terminology® (CPT) or the International Classification of Diseases, version 10, Procedure Classification System (ICD-10-PCS) codes can be combined to count statewide surgical characteristics [10,11]. We designed the current study to determine the distributions of the length of stay among classes of major therapeutic, surgical procedures, based on mapping the performed CPT or ICD-10-PCS codes to Clinical Classification Software (CCS) categories. The objective was to quantify the percentage of cases with major therapeutic procedures unlikely to have postoperative admission for longer than one night.

We selected a threshold of an overnight stay in the hospital for three reasons. First, for patients reliably discharged on the same day of admission (i.e., length of stay = 0 days), considerations related to hospital admission are moot [6]. Second, ambulatory surgery centers represent a distinct class of facilities that are often geographically distant from hospitals where patients can receive care [12]. Third, for patients reliably staying overnight and discharged the next day (i.e., length of stay = 1 day), the impact on the hospital census would be transient. If the hospital reached capacity, then the current day’s elective patients would be kept in the post-anesthesia care unit (PACU) [12] and the next-day elective surgical list would be canceled. The effect on the census would be immaterial if the patients were kept in the PACU overnight and then discharged home the next day. Nelson et al. demonstrated that patients with an expected length of stay ≤1 day who were held in the PACU overnight had a smaller difference between their expected and actual lengths of stay compared to patients not staying in the PACU overnight (−0.096 days, 95% CI −0.30 to −0.031 days) [13].

The primary goal of this study was to provide the post-procedure length of stay data to public health policymakers at the governmental level to better inform their decisions with respect to allowing patients to receive elective surgical care during the COVID-19 pandemic.

## Materials and methods

The University of Miami Institutional Review Board determined on July 13, 2020, that this research does not meet the regulatory definition of human subjects research.

Data sources

We obtained from Florida Health publicly available data for inpatient hospitalizations and ambulatory surgical procedures between January 1, 2018, and December 31, 2018 [14] subject to a data use agreement dated May 28, 2019. These data included every surgical case at every non-federal hospital in Florida [15]. To identify whether an ICD-10-PCS code was for a major therapeutic procedure (i.e., procedure class = 4), we used the “Procedure Classes for ICD-10-PCS” file from the Healthcare Cost and Utilization Project [16]. To determine if a CPT code was for a major therapeutic procedure, we determined if the associated surgery flag field for the code had a value of “narrow.” For mapping CPT procedure codes (the taxonomy used in the ambulatory database) to the corresponding CCS category, we used the “2019 CCS-Services and Procedures Software” [17]. For mapping the ICD-10-PCS codes (the taxonomy used in the inpatient database) to the relevant CCS category, we used the “CCS for ICD-10-PCS Procedures, v2020.1” crosswalk files from the Healthcare Cost and Utilization Project [18]. To determine which of the listed ambulatory procedures was primary, we used the “April 2018 Physician Fee schedule from the Centers for Medicare and Medicaid Services” to map the CPT code to the work relative value units and percentage attributed in intraoperative care [19]. This approach for identification of the primary procedure for cases was used previously for statewide analyses of (a) surgeon cases per day on dates with at least one case and (b) growth in surgeon cases per week from one year to another [9,10].

Elective case inclusion criteria for major therapeutic procedures

For each of the inpatient admissions, the ICD-10-PCS code for the performed primary procedure related to the reason for the hospitalization was identified along with the length of stay in days, the offset in days from admission to when the procedure was performed, emergency room charges, the admission priority of the admission, and the location source of the admission. Elective major therapeutic procedures were identified [20] by the ICD-10-PCS code having a procedure class = 4, performed on the day of admission (i.e., 0 days), having no emergency room charges for the admission, and where the admission priority was not listed as urgent or emergent. (There were 58 patients whose admission source was listed as the emergency room, no emergency room charges, and an admission priority of elective; these patients were included.) Thus, from the inpatient database, we included only non-hospitalized patients who had a scheduled major therapeutic procedure on the day of admission. For the studied 649,962 cases, the primary ICD-10-PCS code was mapped to the relevant CCS category.

For all ambulatory cases, the database included the CPT codes for all procedures performed during the encounter, but, among these, the primary procedure was not identified. We, therefore, inferred which of the major therapeutic CPT codes was primary. After eliminating all CPT codes that were not for a major therapeutic procedure, we determined the primary procedure by determining the CPT with the largest value of operative work (i.e., work relative value units × percent attributed to the OR). We then mapped that CPT code to the relevant CCS category. None of the cases had a tie for operative work that mapped to different CCS categories. We considered all 1,202,429 ambulatory cases to be elective. The ambulatory file included a disposition code indicating if the patient was transferred to a hospital rather than being discharged home or to a non-hospital location (e.g., a nursing home). Ambulatory surgery without hospital admission was counted as having a hospital length of stay of 0 days. If the patient was transferred to a hospital from the ambulatory surgery center, we inferred that the length of stay was >1 day.

Inpatient surgery accounted for 649,962 cases, and ambulatory surgery accounted for 1,202,429 cases.

Calculations

From the combined inpatient and ambulatory cases, we calculated the percentage of cases where the length of stay was ≤1 day. This included patients who were discharged on the day of surgery (i.e., 0-day length of stay) or who stayed overnight (e.g., observation status, extended recovery, planned one-day inpatient admission). For each CCS category, we calculated the percentage of cases for that CCS where the length of stay was ≤1 day. We determined the Clopper-Pearson conservative 95% one-sided upper and lower confidence limits (UCL, LCL, respectively) for the percentage of ambulatory patients transferred to a hospital from the ambulatory surgery center and for the percentage of patients with a length of stay ≤1 day, respectively.

Our study was a descriptive analysis of the 1,852,391 elective surgical cases during 2018 in Florida in which the primary procedure was an elective, major therapeutic procedure; thus, no power analysis was performed.

## Results

**Figure 1:**
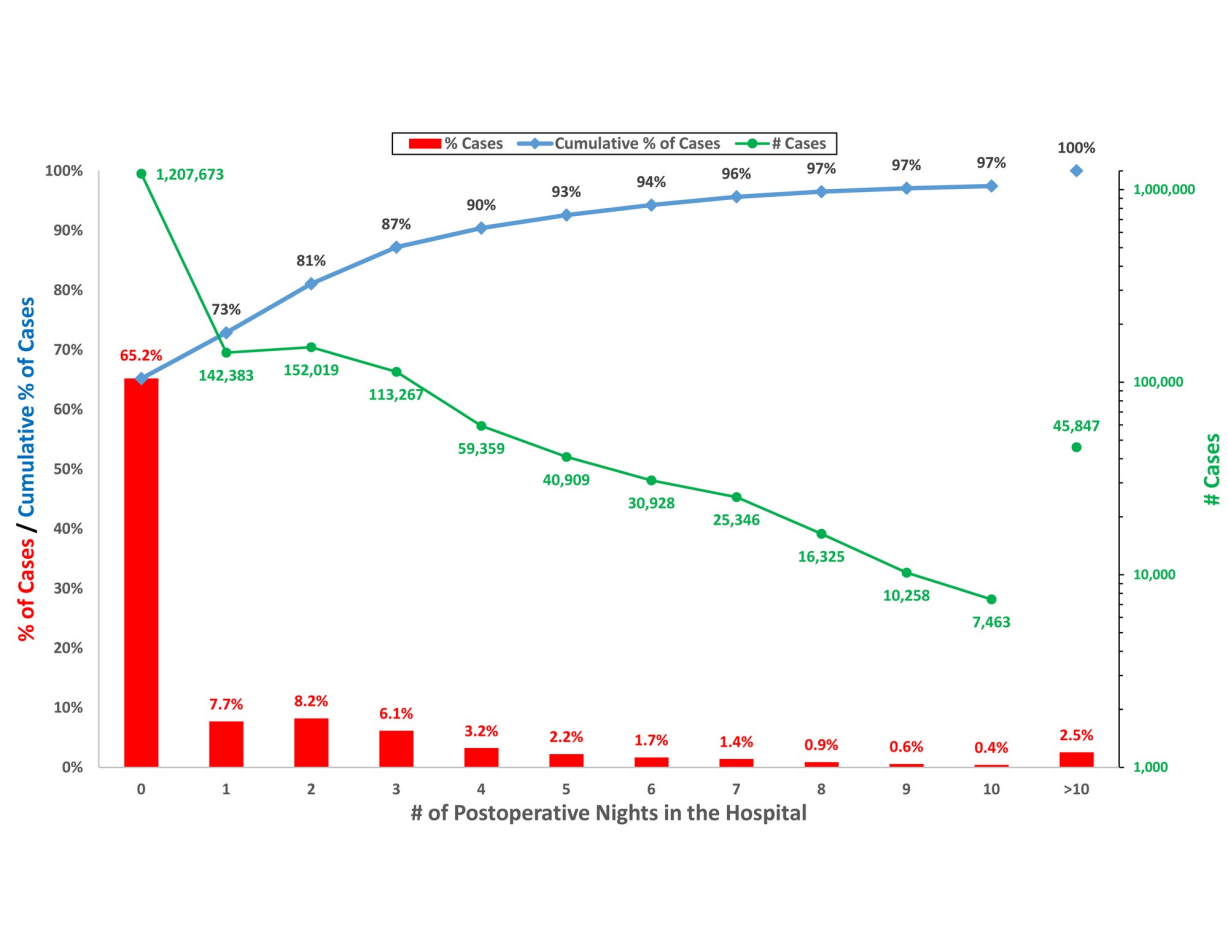
Distribution of postoperative length of stay following all elective, major therapeutic surgical procedures in the state of Florida in 2018. The red bars indicate the percentages of the 1,852,391 cases for each listed length of stay. The blue line represents the cumulative distribution of the lengths of stay. The green line is the count of cases for each of the listed lengths of stay. A discharge on the day of surgery corresponds to 0 nights in the hospital (length of stay = 0 days), while an overnight stay would correspond to a one-day length of stay.

Among the 1,852,391 elective cases that included a major therapeutic procedure, 65.2% (95% LCL = 65.1%) of cases had a length of stay of 0 days and 72.9% (95% LCL = 72.8%) had stay ≤1 day (Figure 1). These were slight underestimates because they excluded 0.051% of the 1,202,429 ambulatory surgery cases where the patient was transferred, following surgery, to a hospital (95% UCL = 0.055%).

Table 1 provides a list of the 101 CCS categories for elective, major therapeutic procedures, and with at least 100 patients having a length of stay of ≤1 day. There were 38 different CCS categories for which at least 95% of patients had a length of stay of ≤1 day (column 5). Such cases would be potentially suitable for scheduling even in the context of a hospital census constraint. There were 28 CCS codes that identified 80% of the patients who were discharged with a length of stay ≤1 day, with multiple surgical specialties represented in this list.

**Table 1:** Proportions of cases with a length of stay of 0 or 1 day among all n = 1,845,011 elective major therapeutic cases performed in Florida in 2018, stratified by CCS category. CCS, Clinical Classifications software; OR, operating room, CNS: central nervous system, GI: gastrointestinal.

CCS	Cumulative proportion of the 1,350,056 cases with length of stay ≤ 1 day	Percentage of the 1,852,391 cases that were of the listed CCS	Percentage of the 1,852,391 cases that were of the listed CCS and with length of stay ≤ 1 day	Less than 5% of the cases in the listed CCS with length of stay >1 day (i.e., suitable for scheduling)	CCS description	Description of the most common primary procedure in the listed CCS in Florida
15	21.9%	16.0%	100.0%	Yes	Lens and cataract procedures	Extracapsular cataract removal with insertion of intraocular lens prosthesis, manual or mechanical technique
160	28.4%	4.9%	95.7%	Yes	Other therapeutic procedures on muscles and tendons	Arthroscopy, shoulder, surgical; with rotator cuff repair
162	32.9%	3.9%	82.7%	No	Other OR therapeutic procedures on joints	Arthroscopy, shoulder, surgical; debridement, extensive
151	36.3%	2.5%	99.9%	Yes	Excision of semilunar cartilage of the knee	Arthroscopy, knee, surgical; with meniscectomy including debridement/shaving of articular cartilage
175	39.5%	2.6%	87.5%	No	Other OR therapeutic procedures on the skin and breast	Tissue grafts, other
6	41.9%	1.9%	91.8%	No	Decompression peripheral nerve	Neuroplasty and/or transposition; median nerve at the carpal tunnel
85	44.3%	1.8%	98.5%	Yes	Inguinal and femoral hernia repair	Laparoscopy, surgical; repair initial inguinal hernia
124	46.4%	2.9%	55.0%	No	Hysterectomy, abdominal, and vaginal	Laparoscopy, surgical, with total hysterectomy, for uterus 250 g or less; with the removal of tube(s) and/or ovary(s)
14	48.6%	1.6%	100.0%	Yes	Glaucoma procedures	Iridotomy/iridectomy by laser surgery
172	50.7%	1.6%	93.4%	No	Skin graft	Adjacent tissue transfer or rearrangement, forehead, cheeks, chin, mouth, neck, axillae, genitalia, hands and/or feet; defect 10 sq cm or less
84	52.7%	1.6%	92.3%	No	Cholecystectomy and common duct exploration	Laparoscopy, surgical; cholecystectomy
142	54.7%	1.7%	85.6%	No	Partial excision bone	Arthroscopy, shoulder, surgical; distal claviculectomy including distal articular surface
23	56.6%	1.4%	99.8%	Yes	Myringotomy	Tympanostomy (requiring placement of ventilating tube), general anesthesia
86	58.6%	1.7%	81.9%	No	Other hernia repair	Repair umbilical hernia, age 5 years or older; reducible
152	60.5%	3.5%	39.0%	No	Arthroplasty knee	Arthroplasty, knee, condyle, and plateau; medial and lateral compartments with or without patella resurfacing
158	62.4%	4.7%	29.2%	No	Spinal fusion	Arthrodesis, anterior interbody, including disc space preparation, discectomy, osteophytectomy, and decompression of spinal cord and/or nerve roots; cervical below C2
30	64.1%	1.3%	98.1%	Yes	Tonsillectomy and/or adenoidectomy	Tonsillectomy and adenoidectomy; younger than age 12
166	65.9%	1.3%	99.2%	Yes	Lumpectomy, quadrantectomy of breast	Mastectomy, partial
161	67.5%	1.3%	87.5%	No	Other OR therapeutic procedures on bone	Removal of implant; deep
19	69.0%	1.1%	99.7%	Yes	Other therapeutic procedures on eyelids, conjunctiva, cornea	Excision or transposition of pterygium; with graft
61	70.5%	1.6%	67.8%	No	Other OR procedures on vessels other than head and neck	Revascularization, endovascular, open or percutaneous, femoral, popliteal artery(s), unilateral; with atherectomy
67	71.9%	1.3%	84.6%	No	Other therapeutic procedures, hemic and lymphatic system	Biopsy or excision of lymph node(s); open, deep axillary node(s)
48	73.3%	1.0%	91.7%	No	Insertion, revision, replacement, removal of cardiac pacemaker or cardioverter/defibrillator	Insertion or replacement of permanent implantable defibrillator system, with transvenous lead(s), single or dual chamber
33	74.5%	1.1%	83.4%	No	Other OR therapeutic procedures on nose, mouth and pharynx	Submucous resection inferior turbinate, partial or complete, any method
118	75.8%	0.9%	98.0%	Yes	Other OR therapeutic procedures, male genital	Laser vaporization of prostate, including control of postoperative bleeding, complete
3	77.0%	1.1%	85.2%	No	Laminectomy, excision intervertebral disc	Laminotomy (hemilaminectomy), with decompression of nerve root(s), including partial facetectomy, foraminotomy and/or excision of herniated intervertebral disc; one interspace, lumbar
153	78.2%	2.2%	39.8%	No	Hip replacement, total and partial	Arthroplasty, acetabular, and proximal femoral prosthetic replacement, with or without autograft or allograft
20	79.3%	0.8%	99.7%	Yes	Other intraocular therapeutic procedures	Vitrectomy, mechanical, pars plana approach; with the removal of internal limiting membrane of retina, includes, if performed, intraocular tamponade
28	80.4%	0.8%	99.8%	Yes	Plastic procedures on nose	Septoplasty or submucous resection, with or without cartilage scoring, contouring or replacement with graft
143	81.3%	0.7%	100.0%	Yes	Bunionectomy or repair of toe deformities	Correction, hammertoe
132	82.1%	0.7%	90.1%	No	Other OR therapeutic procedures, female organs	Laparoscopy, surgical, colpopexy
147	82.9%	0.7%	81.6%	No	Treatment, fracture, or dislocation of lower extremity (other than hip or femur)	Open treatment of distal fibular
9	83.6%	0.8%	69.3%	No	Other OR therapeutic nervous system procedures	Insertion or replacement of peripheral or gastric neurostimulator pulse generator or receiver, direct or inductive coupling
154	84.4%	0.9%	61.4%	No	Arthroplasty other than hip or knee	Arthroplasty, glenohumeral joint; total shoulder
74	85.1%	1.0%	53.3%	No	Gastrectomy, partial and total	Gastrectomy, partial, distal; with Roux-en-Y reconstruction
119	85.8%	0.7%	75.3%	No	Oophorectomy, unilateral and bilateral	Laparoscopy, surgical; with the removal of adnexal structures
148	86.5%	0.6%	81.1%	No	Other fracture and dislocation procedure	Open treatment of metacarpal fracture, single, includes internal fixation, when performed, each bone
113	87.1%	0.5%	92.7%	No	Transurethral resection of prostate	Transurethral electrosurgical resection of prostate, including control of postoperative bleeding, complete
57	87.7%	0.5%	99.9%	Yes	Creation, revision, and removal of arteriovenous fistula or vessel-to-vessel cannula for dialysis	Arteriovenous anastomosis, open; direct, any site
51	88.4%	0.7%	68.6%	No	Endarterectomy, vessel of head and neck	Thromboendarterectomy, including patch graft, if performed; carotid, vertebral, subclavian, by neck incision
10	89.0%	0.5%	90.0%	No	Thyroidectomy, partial or complete	Thyroidectomy, total or complete
106	89.6%	0.4%	99.9%	Yes	Genitourinary incontinence procedures	Sling operation for stress incontinence
129	90.1%	0.4%	99.6%	Yes	Repair of cystocele and rectocele, obliteration of vaginal vault	Combined anteroposterior colporrhaphy
12	90.6%	0.5%	79.5%	No	Other therapeutic endocrine procedures	Parathyroidectomy or exploration of parathyroid(s)
169	91.1%	0.4%	100.0%	Yes	Debridement of wound, infection, or burn	Debridement, muscle, and/or fascia; first 20 sq cm or less
145	91.6%	0.4%	98.3%	Yes	Treatment, fracture, or dislocation of radius and ulna	Open treatment of distal radial extra-articular fracture or epiphyseal separation, with internal fixation
167	92.0%	0.4%	83.4%	No	Mastectomy	Mastectomy, simple, complete
114	92.5%	0.5%	61.4%	No	Open prostatectomy	Cryosurgical ablation of the prostate
21	92.9%	0.3%	99.5%	Yes	Other extraocular muscle and orbit therapeutic procedures	Strabismus surgery, recession, or resection procedure; 1 horizontal muscle
121	93.3%	0.3%	98.9%	Yes	Ligation of fallopian tubes	Laparoscopy, surgical; with fulguration of oviducts
80	93.6%	0.3%	92.2%	No	Appendectomy	Laparoscopy, surgical, appendectomy
96	94.0%	1.1%	22.5%	No	Other OR lower GI therapeutic procedures	Placement of seton
150	94.3%	0.3%	96.6%	Yes	Division of joint capsule, ligament, or cartilage	Capsulotomy; metatarsophalangeal joint, with or without tenorrhaphy, each joint
87	94.7%	0.2%	99.9%	Yes	Laparoscopy	Unlisted laparoscopy procedure; abdomen, peritoneum, and omentum
144	95.0%	0.3%	82.4%	No	Treatment, facial fracture, or dislocation	Fracture nasal inferior turbinate(s), therapeutic
94	95.3%	0.8%	25.6%	No	Other OR upper GI therapeutic procedures	Laparoscopy, surgical, esophagogastric fundoplasty
59	95.5%	0.3%	62.0%	No	Other OR procedures on vessels of head and neck	Transcatheter placement of intravascular stent(s), cervical carotid artery, open or percutaneous, including angioplasty, when performed, and radiological supervision and interpretation; with distal embolic protection
164	95.8%	0.2%	88.0%	No	Other OR therapeutic procedures on musculoskeletal system	Graft; ear cartilage, autogenous, to nose or ear
43	96.0%	1.8%	9.8%	No	Heart valve procedures	Transcatheter pulmonary valve implantation, percutaneous approach
16	96.3%	0.2%	99.7%	Yes	Repair of retinal tear, detachment	Vitrectomy, mechanical, pars plana approach; with endolaser panretinal photocoagulation
112	96.5%	0.6%	25.4%	No	Other OR therapeutic procedures of urinary tract	Aspiration of bladder; with insertion of suprapubic catheter
26	96.6%	0.2%	83.5%	No	Other therapeutic ear procedures	Cochlear device implantation, with or without mastoidectomy
157	96.8%	0.2%	58.6%	No	Amputation of lower extremity	Amputation, toe; metatarsophalangeal joint
170	97.0%	0.1%	100.0%	Yes	Excision of skin lesion	Endoscopic plantar fasciotomy
125	97.1%	0.2%	53.4%	No	Other excision of cervix and uterus	Laparoscopy, surgical, myomectomy, excision; 1 to 4 intramural myomas with total weight of 250 g or less and/or removal of surface myomas
99	97.3%	0.8%	14.0%	No	Other OR gastrointestinal therapeutic procedures	Unlisted laparoscopic procedure; liver
42	97.4%	0.3%	28.6%	No	Other OR therapeutic procedures on respiratory system	Laryngoscopy, direct, operative, with excision of tumor and/or stripping of vocal cords or epiglottis
22	97.5%	0.1%	99.9%	Yes	Tympanoplasty	Tympanoplasty without mastoidectomy, initial or revision; without ossicular chain reconstruction
109	97.7%	0.1%	86.0%	No	Procedures on the urethra	Meatotomy, cutting of meatus; except infant
120	97.8%	0.1%	77.4%	No	Other operations on ovary	Follicle puncture for oocyte retrieval, any method
49	97.9%	0.5%	16.6%	No	Other OR heart procedures	Insertion of ventricular assist device, implantable intracorporeal, single ventricle
123	98.0%	0.2%	34.6%	No	Other operations on fallopian tubes	Tubotubal anastomosis
60	98.1%	0.2%	30.9%	No	Embolectomy and endarterectomy of lower limbs	Excision of infected graft; extremity
104	98.2%	0.5%	16.6%	No	Nephrectomy, partial, or complete	Laparoscopy, surgical; partial nephrectomy
141	98.3%	0.1%	95.9%	Yes	Other therapeutic obstetrical procedures	Cerclage of cervix, during pregnancy; vaginal
24	98.4%	0.1%	98.6%	Yes	Mastoidectomy	Tympanoplasty with mastoidectomy; with intact or reconstructed wall, without ossicular chain reconstruction
17	98.5%	0.1%	100.0%	Yes	Destruction of lesion of retina and choroid	Destruction of localized lesion of retina, 1 or more sessions; photocoagulation
13	98.6%	0.1%	99.4%	Yes	Corneal transplant	Keratoplasty (corneal transplant); penetrating
171	98.7%	0.1%	84.3%	No	Suture of skin and subcutaneous tissue	Secondary closure of surgical wound or dehiscence, extensive or complicated
78	98.7%	1.1%	5.0%	No	Colorectal resection	Excision of rectal tumor, transanal approach; not including muscularis propria
53	98.8%	0.1%	99.9%	Yes	Varicose vein stripping, lower limb	Stab phlebectomy of varicose veins, one extremity; 10-20 stab incisions
174	98.9%	0.1%	99.9%	Yes	Other non-OR therapeutic procedures on skin and breast	Insertion or replacement of cranial neurostimulator pulse generator or receiver, direct or inductive coupling; with connection to a single electrode array
176	98.9%	0.1%	65.5%	No	Other organ transplantation	Ocular surface reconstruction; amniotic membrane transplantation, multiple layers
117	99.0%	0.0%	100.0%	Yes	Other non-OR therapeutic procedures, male genital	Placement of interstitial device(s) for radiation therapy guidance, prostate, single, or multiple
36	99.1%	0.8%	5.4%	No	Lobectomy or pneumonectomy	Removal of lung, other than pneumonectomy; single segment
2	99.1%	0.1%	54.6%	No	Insertion, replacement, or removal of extracranial ventricular shunt	Replacement or revision of cerebrospinal fluid shunt, obstructed valve, or distal catheter in shunt system
244	99.2%	0.0%	100.0%	Yes	Gastric bypass and volume reduction	Laparoscopy, surgical, gastric restrictive procedure; removal of adjustable gastric restrictive device, and subcutaneous port components
101	99.2%	0.1%	54.5%	No	Transurethral excision, drainage, or removal urinary obstruction	Cystourethroscopy, with the removal of foreign body, calculus, or ureteral stent from urethra or bladder; complicated
55	99.3%	0.3%	14.3%	No	Peripheral vascular bypass	Bypass graft, with other than vein; femoral-femoral
146	99.3%	0.2%	18.6%	No	Treatment, fracture, or dislocation of hip and femur	Treatment of slipped femoral epiphysis; by single or multiple pinning, in situ
63	99.4%	0.0%	98.4%	Yes	Other non-OR therapeutic cardiovascular procedures	Insertion of intravascular vena cava filter, endovascular approach including vascular access, vessel selection, and radiological supervision and interpretation, intraprocedural roadmapping, and imaging guidance, when performed
149	99.4%	0.0%	99.8%	Yes	Arthroscopy	Arthroscopy, shoulder, surgical; with the removal of loose body or foreign body
1	99.5%	0.2%	13.4%	No	Incision and excision of CNS	Revision or removal of intracranial neurostimulator electrodes
95	99.5%	0.0%	100.0%	Yes	Other non-OR lower GI therapeutic procedures	Destruction of lesion(s), anus, extensive
159	99.5%	0.0%	99.8%	Yes	Other diagnostic procedures on musculoskeletal system	Arthrotomy, radiocarpal or midcarpal joint, with exploration, drainage, or removal of foreign body
134	99.6%	2.5%	0.9%	No	Cesarean section	Cesarean delivery only;
29	99.6%	0.0%	99.8%	Yes	Oral and dental Services	Alveoloplasty, each quadrant
103	99.6%	0.1%	27.7%	No	Nephrotomy and nephrostomy	Nephrolithotomy; removal of calculus
90	99.7%	0.2%	10.8%	No	Excision, lysis peritoneal adhesions	Enterolysis (freeing of intestinal adhesion)
168	99.7%	0.0%	51.1%	No	Incision and drainage, skin and subcutaneous tissue	Incision and drainage, deep abscess or hematoma, soft tissues of neck or thorax;
56	99.7%	0.1%	14.4%	No	Other vascular bypass and shunt, not heart	Venous anastomosis, open; portocaval

## Discussion

Our results show that even in the face of constraints imposed by a high hospital census, such as for many hospitals currently dealing with an influx of COVID-19 inpatients, many categories of major therapeutic elective procedures could be performed without compromising hospital capacity. For most of these cases (65%), patients would be sent home on the same day of admission, making consideration of hospital census irrelevant. Even when an overnight stay is needed, ambulatory patients could be recovered in the PACU, again not affecting the inpatient census [1]. We identified many individual CCS categories, comprising multiple surgical specialties, in which the likelihood of hospital admission for more than an overnight stay is less than 5%. Thus, statewide or provincial policies should not create a carve-out policy for elective surgery such as “ophthalmology only.” Rather, policies can reasonably be based on the cases with a substantial probability that the patient would have at most an overnight stay [6]. Our results show that our results apply to at least 72.8% of cases.

Previously, we studied how to combine procedures done in the inpatient and outpatient setting for purposes of counting cases by surgeon [9,10] and in the current study to map to their common CCS category. Thus, there was independence regarding whether the same procedure was scheduled to be done in an ambulatory surgery center or in a hospital, with an expected discharge on the day of surgery or the next day. For policymakers and hospital administrators, the implication of our study is that when considering the limitation of elective surgical procedures in the face of the current COVID-19 pandemic, the driving force should not be to forbid “elective” cases or only to allow cases for specific specialties. Rather, they should consider the potential risk of patients requiring postoperative admission for more than one night [6]. Such flexibility might require hospitals to change their policies regarding the overnight boarding of patients in the PACU. Still, as Nelson et al. demonstrated, there was a reduction in the length of stay when patients with an expected length of stay ≤1 day were kept in the PACU overnight [13]. We think the likely reason for this finding was that there was a motivation to discharge patients early the next morning from the PACU to make room for the next day’s surgical schedule. We previously demonstrated, also using administrative data from Florida, that early morning hospital discharges of inpatients from acute care hospitals are uncommon, occurring in only 13.0% (0.28% standard error) of cases and unchanged between 2010 and 2018 [21].

For minor diagnostic and minor therapeutic procedures, the frequency of hospital admission is so small that there would be no reason to limit surgeries based on hospital census considerations. We were not able to analyze major diagnostic procedures because it was not possible to unambiguously determine from the database if the case utilized operating room services, including care by anesthesiology practitioners. The presence of anesthesia charges does not reliably identify who received anesthesia services. For example, anesthesia charges would be listed if a proceduralist administered a local anesthetic.

We emphasize that consideration of hospital census and the expected length of stay are not the only limitations that need to be considered when deciding if elective major therapeutic surgery can proceed during the current COVID-19 pandemic. Personal protective equipment is needed to take care of these patients, and if those supplies are in very short supply, it might not be possible to run a full elective surgical schedule [7]. If the perioperative staff pool is depleted either through contracting COVID-19, being under quarantine, or diverted to take care of inpatients, the elective surgical schedule might need to be reduced [6,7]. Finally, if a patient has serious comorbidities or experienced previous complications (e.g., severe postoperative vomiting) likely to result in postoperative hospitalization, he or she may not be a candidate for an elective procedure in the context of census constraints created by the pandemic.

Our study’s public policy implications extend beyond that related to the COVID-19 pandemic, with applicability to other public health crises in which inpatient hospital resources may be strained. For example, our results can be useful in the event of an especially virulent influenza season or another viral pandemic distinct from that caused by SARS-CoV-2.

Limitations

First, the data used in this study were from a single state, so the generalizability of the findings to other states in the United States might be limited. However, Florida is a large state with a diverse population and offering a full complement of surgical services. In 2006, ambulatory surgery procedures in the USA were 62% of the ambulatory plus inpatient total [9]. The fact that in 2018 the percentage of ambulatory surgery in Florida would be greater (65%), but not dramatically different, is reasonable. Thus, we think that the findings are likely applicable to other regions. Second, there is no mapping of patients between the ambulatory and inpatient cases, so it was not possible to determine the lengths of stay of patients who were transferred from an ambulatory surgery center to a hospital, presumably due to a complication. However, the percentage of such patients was extremely low (approximately 0.05%), and we deliberately biased the study in counting such patients as having a length of stay of >1 day. Thus, the absence of the actual length of stay could not have substantively altered our results. Finally, the data available did not allow an analysis of delayed hospital admission following same-day discharge because there were no patient-level identifiers to track the occurrence of such events. To the extent that delayed readmission rates are known for a given procedure, modification of the list of acceptable procedures might be necessary.

## Conclusions

There are a large and diverse number of CCS categories comprising elective, major therapeutic surgical procedures that reliably will have hospital lengths of stay of ≤1 day. Because many patients need surgery to improve their quality of life and to avoid deterioration in medical conditions, even during a global pandemic, public policy leading to an arbitrary restriction of surgery based solely on whether the procedure is “elective” is unwarranted. Rather, policymakers should consider the likely impact of performing the primary surgical procedure, based on its CCS category, on the patient requiring hospital admission and the extent to which local hospital inpatient beds are constrained. Because the COVID-19 pandemic may remain widespread in the United States for a substantial period, the indefinite postponement of elective, major therapeutic surgery is not a viable alternative. The approach we describe is one aspect of a strategy to allow the vast majority of surgical procedures to be performed while preserving an adequate amount of hospital resources to provide care for COVD-19 patients.
